# American Academy of Dental Science

**Published:** 1891-03

**Authors:** 

**Affiliations:** American Academy of Dental Science


### AMERICAN ACADEMY OF DENTAL SCIENCE.

The regular monthly meeting of the American Academy of Dental Science was held in the Boston Medical Library Association rooms, December 3, 1890.

President Seabury in the chair.

The paper of the evening was read by Dr. George F. Eames; subject, "The Obtunding of Sensitive Dentine." (For Dr. Eames's paper, see page 158.)

*Dr. Fillebrown.*—I have been much interested in the paper, My experience with hot air or cold spray has not been extensive. and this because the experience which I have had with them has not been very favorable. Their application has inflicted too much pain. This is the case with other obtundents, as phosphoric acid, and in particular chloride of zinc. These generally produce considerable pain, but are effective. With cocaine I have had more uniform and satisfactory success than with any other substance I have used. I have had marked successes with it in many cases where one would think it would hardly accomplish anything,—for instance, in buccal or labial cavities where the rubber dam is not applied, but a napkin is laid upon the gum and the cavity made moderately dry. In these cases, without the use of alcohol or hot air for drying, but simply by removing moisture with absorbent cotton, and laying on a piece of cotton saturated with cocaine and allowing it to remain for a minute or two, a wonderful change takes place. The patients themselves will notice it and speak of it. It will not always palliate, but it will do so in a larger proportion of cases than any other agent that I have ever used. I wish also to say amen to the use of the anæsthetic power of ether for obtunding sensitive dentine. I have patients who for a long time have used it whenever they have had an operation performed, and they will not allow me to do anything to their teeth until they get their dose of ether. By taking it to the first stage of anæsthesia, I can cut away and get the teeth into good shape, and the patients do not suffer at all. But for the prejudice against ether on the part of so many patients,

I would use it a great deal oftener than I do. If it were not for the prejudice that exists against chloroform, I would use it instead of ether. Chloroform has a pleasant odor, and a very small quantity is effective. Its vapor has not the irritating quality of ether vapor, and it will obtund the sensitiveness of dentine with a less degree of anæsthesia than will ether. I have one patient, a dentist, for whom I have performed operations for a number of years. His teeth are very sensitive indeed, and he invariably takes a little chloroform on a napkin and inhales it; and while he is still conscious and perfectly rational, and can talk and tell me what is going on, he can have the operation performed with perfect ease. I think this matter is worth our attention. The method is perfectly reasonable, and I wish more would try it. Recently I underwent a minor surgical operation. In order not to feel the pain, I took some chloroform on a napkin and inhaled it until I felt all right, and then said to the operator to go ahead. I retained my senses and held control of the chloroform and of myself. I knew what was going on, talked about it all the while, and told the operator that he was not hurting me. The operation was soon over, and I felt all right in a few minutes. I think the inhalation of anæsthetics to obtund sensitive dentine a practical idea to entertain, and it is worth our while to give it more attention.

*Dr. Williams.*—There are several points in the paper with which I fully agree. I was glad to hear the rationale of the sensitiveness of dentine so thoroughly elucidated. The matter of obtunding sensitive dentine of course comes to us in a practical way. Some time, perhaps, I will allude in a paper to a system I devised for obtunding sensitive dentine before ether was used. It was not a complicated or dangerous process. It consisted in allowing a simple solution of chloride of lime to remain a short time in the cavity. Then with a sharp instrument, and the sharpness of the instrument was an important factor, you could cut where you could not touch before. This method I have used to a considerable extent since. For the general obtunding of sensitive dentine I have found lime-water effective. Bicarbonate of soda often does the same thing, though more disagreeable to the taste. If patients will simply rinse the mouth with dilute lime-water, not strong enough to be caustic, it is a good application to sensitive dentine.

Speaking of ether, my preceptor, Dr. Keep, was rather intimately connected with some of its promoters, and I had an opportunity to see how it was received by the public. At first it was used very extensively; patients demanded it,—they thought that the day of the millennium had come, that they could take ether while undergoing any operation and be free from all discomfort. There were two or three who insisted on taking ether at once as soon as they sat down, before having the teeth examined, to be unconscious of what was needed to be done. Of course there were very great objections to that, and we often had to use strong argument to convince them that it was not wise to use it so freely. When I opened an office of my own, I discarded ether largely because of the disagreeable odor in the office, which I carried out in my clothes, and which patients carried around with them for several days after it was used. In some cases, too, nausea was produced, which was objectionable.

When chloroform began to be substituted for ether, one or more cases at the hospital resulted fatally, and I have no doubt that if we had not by habit been so very careful in the application of sulphuric ether in Dr. Keep's office, there would have been fatal results from the use of chloroform. We had several cases of collapse, in which there was cessation of breathing in some instances and cessation of heart-beat in others, and at times it was only by the greatest vigilance that we succeeded in bringing such cases around, and most of them were not the result of a heavy dose. I remember one case as showing the idosyncrasy of some constitutions with regard to the influence of ether. A lady was overcome by ether which was merely unstopped in a bottle six or eight feet from her,—the simple "intimation" of the odor checked the circulation. That, of course, was an unusual thing. I remember a scare which one of our older physicians had in my office. His brother was a patient of mine, and required the extraction of two or three roots which were rather irritating, and he proposed to take ether. They were using chloric ether at that time as a modification of chloroform, and the patient asked if he might have his brother come in and give it to him. I said certainly, I should be very glad to have him relieve me of that part of the care. So the physician gave it, and a few seconds after the extraction the patient collapsed. He fell back and ceased to breathe, his eyes were glazed, and he grew purple in the face, and no pulse could be felt. The physician turned as pale as the brother was purple, and called for ammonia. I told him there was no time for that, there was not a second to be lost, we must get him to breathing *at once*, and I commenced the bellows action of the chest and kept it up steadily, not even stopping to put him into position for working the arms. Very soon his face began to brighten, and he recovered. There is a value to this bellows action, I think, that is not often apprehended. No time should be lost,—not even to get your appliances; the action of breathing forces the heart to act so that the circulation is not fatally stopped.

I agree that aside from these there are other ways of obtunding sensitive dentine, and the use of cocaine, of which the essayist speaks so highly, certainly is of great value. I think that it has been with me the most satisfactory ready obtundent I have used, although I have been disappointed in some cases of very dense dentine. Even those cases generally would be affected; and instead of giving three or four minutes for it to take effect, I would seal up the cocaine in the cavity and go on with some other work for ten or fifteen minutes or more. Sometimes in applying it to the outside of a tooth, I put a piece of paraffine paper over it to prevent its washing away, and let it rest. Time renders the application more effective. In its application to soft dentine, by mixing the cocaine with lanoline or agnine its absorption is facilitated.

*Dr. Eddy.*—In connection with this topic I should like to show one of Mr. Small's latest appliances. I do not approve of chloroform; I think it is liable to be fatal, and I do not like ether on account of its odor. This instrument is an improvement over the "steam cooker," as Dr. Cooke calls it, which was shown here at one of our previous meetings. It is much smaller than the old one, and is more easy to handle. It consists of a little cylinder containing a cartridge into which is put some cotton saturated with alcohol. This rubber tube is simply intended to keep the heat from the hands. By heating the bulb here the hot air vaporizes the alcohol, and you get a spray of hot alcohol vapor from this point. As there is no lamp with it there is no danger of any explosion. Mr. Small brought it to me about two months ago, and I have used it somewhat, perhaps not as much as I would wish.

*Dr. Cooke.*—Is Mr. Small selling those appliances?

*Dr. Eddy.*—I understand he is; I believe the price is fifteen dollars.

*Dr. Cooke.*—I saw in one of the British journals lately an illustration of an appliance just like that. It was shown in Berlin by a Mr. Simonis before one of the societies there, the price being a few shillings.

*Dr. Banfield.*—I would like to ask Dr. Eddy if there is anything to make them so expensive?

*Dr. Eddy.*—Not in the material or cost of manufacturing. I should think thirty-five or forty cents ought to make it.

*Dr. Briggs.*—I am very much interested in this paper and have obtained several suggestions from it. I simply want to testify to the method by which I obtund sensitive dentine, which has something of the same principle as the essayist advocates. I first obtund the sensitiveness somewhat with a solution of cocaine, and then follow that with a mixture of carbolic acid and caustic potash to destroy the contents of the tubuli. This treatment is very effective.

Referring to the remarks on ether and chloroform, I think we ought always, in speaking of a subject of that sort to those who have not made a special study of their use, to caution them that they are attended with considerable danger,—especially chloroform. I do not use ether because the odor is so very bad. I have used chloroform many times as spoken of here to-night, but I wish to give to others the very strong caution to remember that the state of incomplete anæsthesia is considered the most dangerous state the patient can be in. At that time the heart is slightly enfeebled by the action of the anæsthetic, and if you cause sufficient pain you may by reflex action stop the heart entirely. So that the state of incomplete anæsthesia is more dangerous than the complete state, where the danger is not to the heart, but to the respiration, the heart beating for several minutes after breathing has ceased, and a prompt means of continuing respiration generally restoring the patient. It is, therefore, important to bear in mind these facts when deciding as to whether you will put a person under complete or incomplete anæsthesia, although ordinarily the pain from a tooth is not sufficient to cause reflex action.

*Dr. Williams.*—From my observations I think Dr. Briggs's remarks would apply to chloroform rather than to sulphuric ether. My experience with the use of partial anæsthesia has been confined to sulphuric ether, and I have found that it gives the patient a sort of "Dutch courage." I sometimes used it in light operations, as the taking out a small root, and have never had any trouble in its use while producing partial anæsthesia. With chloroform I have seen rather dangerous threatenings.

*Dr. Niles.*—There seems to be in the profession a general attention to the subject of sensitive dentine. During the past year five or six improved methods and secret preparations have been advertised or presented to us in one way or another. I am of the opinion, however, that the restrictions of sale are such that professional honor precludes many from using them. The appliance which has been shown here to-night I have been somewhat connected with, and perhaps it is best for me to explain just how I am interested in it.

Last spring, as you know, an appliance was exhibited here which was called the "steam cooker." I experimented with it and met with very fair results, but it was very clumsy and unmanageable. I think it was the first instrument of this kind that Mr. Small made. He afterwards made this modification of the appliance, which he brought to me just as I was about to start for Berlin. He desired me to take the appliance along with me. I asked him how he intended to put it on the market. He told me that he had decided to sell them outright at a fair price, also stating that it was patented in America, France, and England, but not in Germany. It was my opinion, and I think I so expressed it to him, that, if the instrument would do what he claimed for it, the profession would be willing to pay him a fair price—at least ten dollars apiece—for the instruments, but that they would not take hold of them at one hundred, fifty, or even twenty-five dollars, as he proposed. I had only a few moments for conversation with him, and he left to my judgment the sale of the instrument in Germany. After showing the apparatus in Berlin, I was invited to clinic. I did so for three consecutive days, and met with extraordinary success; in no case did it fail of the desired result. I had seven patients, all dentists but one, and all of them assured me that they did not experience the least pain in its use, or in the excavating following its use. It was received with a good deal of enthusiasm; thirty-two or thirty-three gentlemen were anxious to be supplied with them at once. Acting upon the instructions which the inventor gave me before leaving this city, I made arrangements with a dental friend in Belgium to fill orders at ten dollars a piece, forty marks. These orders were filled and the inventor approved my action. I have recently learned that, on account of there being no patent on the invention in Germany, the instrument has been reproduced by manufacturers there so as to be sold for six marks. I would not care to use the instrument nor recommend it if its price were one hundred dollars, or if dentists were to get an instrument free by taking a share of stock in the company. In my opinion, it is as unprofessional to rob the inventor as it is to permit him to rob us by encouraging an exorbitant price. I think he is entitled to some credit for having invented the appliance, and that ten dollars is not a high price for it. If we use the invention, the inventor should be paid. I use the instrument almost daily, and am very much pleased with it. It is not always successful; a great deal depends upon the density of the dentine. But in the teeth of children, if kept dry, it acts very quickly and effectively. With adult teeth the action is slower, and often produces considerable pain before the sensitiveness of the dentine is controlled. An application of a few seconds is often all that is necessary to produce complete insensibility to pain in very sensitive teeth.

*Dr. Ainsworth.*—I should like to ask Dr. Niles if in the use of this instrument or the one that preceded it he has ever had any unpleasant results on the pulp?

*Dr. Niles.*—I have never used it where a tooth was decayed near the pulp, and have never seen any bad results, or known of any, from the use of this instrument.

*Dr. Ainsworth.*—It strikes me that this is a great improvement over the other. I used the other instrument a few times, but with very little satisfaction, and I was informed of a case where there was a good deal of trouble apparently from over-cooking the pulp. That happened at a clinic, but it seems to me this instrument could be handled more easily, and would therefore be free from as much danger.

*Dr. Eames.*—In regard to the steam obtunder exhibited by Dr. Niles, I have not heard it stated here just what it is that obtunds. I suppose, however, its virtue is wholly due to the temperature established in the dentinal fibrils. I have a jeweller's blow-pipe about eight inches long that acts on identically the same principle as the instrument shown here this evening. There is a wick saturated with alcohol enclosed in the pipe. If you heat one end of the tube, the vapor comes forth in a jet. I see nothing new in principle, simply a new use of the principle.

I have the pleasure, through the courtesy of Messrs. Codman & Shurtleff, of showing you their new compressed air-tank, the instrument to which I referred in my paper, and by means of which we can have air on tap at any desired temperature. Here is the air-pump with two cylinders and a gauge. Any desired pressure can be obtained, and just by the touch of the finger the air can be let out at the syringe-pointed nozzle. The air is not warmed in these cylinders, but in a separate air-tight tank connected with this. I did not bring that with me to-night, as I have not had time to complete certain improvements that I am making in it. In order to secure a favorable condition for the absorption of cocaine in the hard, dense teeth of elderly persons, I use more of the warm air. I had a case this afternoon where I used warm air exclusively with good effect, applying a continuous stream for a considerable time.

*Dr. Briggs.*—Would it be possible to warm the air in the tank?

*Dr. Eames.*—Yes, but if you use a very long tube, especially a rubber tube, the air will get cool before it reaches the end, and you do not have the same temperature as in the tank.

*Dr. Briggs.*—Does it not require a pretty large chamber, so as not to allow the air to cool?

*Dr. Eames.*—Not so large as I at first supposed. A tank the size of a quart is sufficient. I could let air very rapidly into the tank through an orifice a quarter of an inch in diameter, and it would become heated as fast as it entered. Small metal tubing heated at one point will warm the air sufficiently, even though the air passes rapidly through it.

*Dr. Fillebrown.*—At what temperature do you use the air?

*Dr. Eames.*—In view of the theory that I have advanced, 105° Fahrenheit should give the best results.

*Dr. Fillebrown.*—I have been using for a long time cocaine and chloroform separately, but I should think that a solution of cocaine in chloroform might answer the purpose just as well.

*Dr. Eames.*—A ten-per-cent. solution makes a very good mixture, and is fairly clear, as you will see by the bottle I have just passed around. I have used this solution only a few times; I am in the habit of using warm air to quite an extent, raising the temperature of the tooth and removing moisture from the dentine. I then use absolute alcohol and then dry the tooth. I next apply the solution of cocaine, and afterwards apply chloroform on cotton to hasten the absorption of the cocaine. This process to be repeated when necessary.

With regard to the state of partial anæsthesia being a condition of danger, it does seem a reasonable conclusion when we remember that the nucleus of the fifth nerve and the nucleus of the pneumogastric are very closely connected. A peripheral injury, therefore, referred to the fifth may be transmitted to the cardiac branch of the pneumogastric and paralyze the heart. Still I have used partial anæsthesia very generally, and have never had any serious results.

As to the injection of cocaine into gum-tissue, to obtain good results these are the requisites: A sharp needle and a good syringe. Inject the solution with considerable force until the tissue turns perfectly white for a considerable distance about the tooth. When this effect is produced in every case the tooth can be removed without pain.

*Dr. Briggs.*—May I ask Dr. Eames how much he injects, and of what strength?

*Dr. Eames.*—Of a four-per-cent. solution I usually use from five to ten minims, and a proportionate amount of the stronger solutions; that is, upon the average patient. There are those who may be susceptible to a less quantity. In the injection of cocaine into gum-tissue I am mindful of these facts: The dose by the stomach is one-eighth grain to three grains; under the skin, two minims of a four-per-cent. solution. In injecting into the gum, however, much is wasted and some is probably carried off by the hemorrhage which follows. We may therefore expect that a comparatively small portion of the dose enters the circulation. It would be a far different matter if the needle should go below the gum into the vascular tissues at the junction of the cheek. In such a case a very sudden and profound effect might be experienced. Provided the whole amount were sure to go into the circulation, two minims of a four-per-cent. solution should be enough for the first injection, in case the patient has never had it used before.

*Dr. Briggs.*—I got constitutional effects the other day from a twenty-per-cent. solution of cocaine passed down around the root of a molar tooth that I wished to scale thoroughly, not using the hypodermic syringe, but simply bathing around the root. The patient seemed to be very susceptible, but of course that is a somewhat stronger solution than one would inject.

*Dr. Williams.*—Cocaine is one of those things that seem to act differently on different constitutions. You cannot make a machine thing of it,—cannot always calculate what the effect will be.

*Dr. Eames.*—I would suggest that every one should know the physiological antagonist of cocaine. Nitrite of amyl is the antidote. The best form for use is the pearls, which are little glass capsules, and are simply broken, crushed in a handkerchief, and inhaled. No one should use cocaine without having the antidote at hand.

*President Seabury.*—I would say that, for the last six years at least, I have been using for sensitive dentine, especially in the teeth of children, a little atomizer with which I spray ether. I feel sure that I can take up my atomizer and obtund the sensitiveness of the tooth and excavate it and get through while you are getting these things ready. In my hands it is very uniform. A half-minute's application of the spray of common sulphuric ether into a cavity is very effective. You have the atomizer right at hand; use it and get through with it, and that's the end of it.

*Dr. Brackett.*—Do you use an ordinary atomizer such as you would use for perfumery, operated by an elastic bulb?

*President Seabury.*—Yes; direct it right into the cavity and spray it there for, say, half a minute, put it down, take up your excavator, and cut off that sensitive layer. It can be used with or without the rubber dam.

*Dr. Stevens.*—I should think the ether would be likely to dissolve the rubber dam.

*President Seabury.*—I haven't noticed that it does; it would hardly do it in so short a time.

*Dr. Taft.*—In excavating around the gums in labial cavities, on the incisors, for instance, you would not use it without protecting the gum, would you? Would it not freeze the gum?

*President Seabury.*—I never freeze the gum. If the margin of the gum begins to turn white I stop,—that is enough. You need not have as much as that, even; it hasn't to be carried to that extent. Any man who uses it will become familiar with it and know exactly when to stop. My rule was, when I first began to use it, to apply it until I saw the margin of the gum begin to turn white; but now, where I do that once, I use it ten times without, and with as much uniformity of result as in any other operation. The beauty of it to me is that it is quickly done and over with.

Subject passed.

*Dr. Eddy.*—I have here a very neat blow-pipe which I found in a jeweller's supply store. I bought it of the Waterbury Brass Company, Providence, and presume some of the jeweller's supply stores here have it. Price, $2.50.

William H. Potter, D.M.D.,
*Editor American Academy of Dental Science.*

